# The Role of Keeving in Modulating Fermentation and the Flavour Profiles of Apple Brandy

**DOI:** 10.3390/biom14101322

**Published:** 2024-10-18

**Authors:** Magdalena Januszek, Paweł Satora, Aneta Pater, Łukasz Wajda

**Affiliations:** 1 Department of Fermentation Technology and Microbiology, Faculty of Food Technology, University of Agriculture in Krakow, Balicka Street 122, 30-149 Kraków, Poland; magdalena.januszek@urk.edu.pl (M.J.); aneta.pater@urk.edu.pl (A.P.); 2 BioLyo Technologies Ltd., Technologiepark-Zwijnaarde 94, 9052 Gent, Belgium; lukasz.wajda8@gmail.com

**Keywords:** defecation, keeving, apple brandies, volatile compounds, sensory analysis

## Abstract

Keeving is the removal of nutrients from apple musts due to their binding to pectin, resulting in a slower fermentation and spontaneous arrest. The aim of this study was to determine the effect of keeving on the chemical composition of fermented apple must and on the volatile profile and sensory analysis of apple brandies. We compared the application of keeving during spontaneous fermentation with fermentation carried out by *Saccharomyces cerevisiae* (SafSpirit HG-1). We evaluated the impact of adding different doses of calcium chloride on various parameters of fermented musts and distillates. Calcium chloride had a greater effect on the ethanol concentration, total extract, and fermentation efficiency than on the type of fermentation used. However, a different phenomenon was observed with respect to the volatiles. The concentration of most of the higher alcohols, acetaldehyde, dodecanal, and geranylaceton, decreased after spontaneous fermentation and increased during the fermentation carried out with *Saccharomyces cerevisiae* SafSpirit HG-1. In general, the application of keeving contributed to a decrease in the concentration of ethyl and methyl esters, but caused an increase in the concentration of all acetate esters and terpenoids. When the amount of nutrients in the environment is limited and starvation occurs, microorganisms use the available nutrients for basic metabolic processes that allow them to survive and limit the formation of side metabolites such as volatiles. However, most of the samples fermented after the faecal depletion achieved high scores for the floral, fruity, and “overall note” parameters in the sensory analysis. This means that this method, carried out with a properly selected yeast strain, could be feasible for the distilling industry.

## 1. Introduction

Keeving is not commonly used in traditional wine and spirit production. It is occasionally used in cider production in France and is called maceration et cuvage. Keeving is the removal of nutrients (a source of nitrogen and carbon for yeast) from apple must. As a result of this method, fermentation slows down and stops spontaneously. Calcium chloride and pectinoesterase are added to improve the dewatering process and to ensure that the amount of calcium is sufficient to form a gel (chapeau brun) [1,2,3]. The demethylation of the linear chain of the homogalacturonan units in pectin allows binding with calcium, which enables gel formation. This stage of the process can occur after 2 to 6 days of fermentation and is activated by the CO_2_ generated at the beginning of fermentation, which allows the gel to rise to the surface of the must [4]. To ensure proper keeving, it is not recommended to use the clarifying agents commonly used in the wine industry, which could cause pectin depolymerisation. As a result of this reaction, there would be no gel formation. According to Lea A. [1], only pectin de-esterification needs to take place. If depolymerisation also occurs, the gel will not form. In addition, the calcium and enzymes should not be added at the same time [1,3].

**Figure 1:**
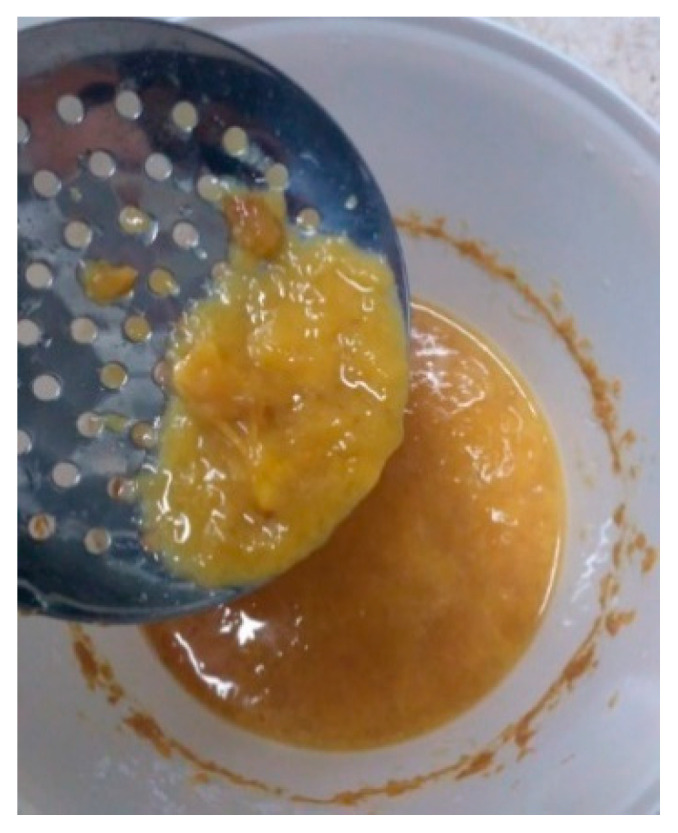
Dissolution of the gel during the fermentation process after keeving.

After a few days of fermentation, the gel should be present on the surface (Figure 1). When the thickness of the gel is 2–3 cm, it is recommended to decant the clear liquid. Failure to do so will cause the gel to disintegrate and will force the keeving process to be repeated (Figure 1). As a result of the racking process, clear must can be obtained and the slow fermentation process continues. The slow fermentation rate is associated with the removal of nutrients from the liquid and the removal of the yeast biomass. Almost 50% of the pectin and nutrients are trapped in the gel and sediment remaining after decanting the fermenting must [3].

In the case of traditional decantation, fermentation takes place spontaneously. It is assumed that a lower concentration of nutrients in the must, especially amino acids, reduces the concentration of higher alcohols formed. This group of substances could form after the decarboxylation and deamination of the exogenous amino acids. A significant reduction in the rate of fermentation could increase the formation of esters. Calcium chloride and pectinoesterase are added to improve defecation and provide a sufficient calcium concentration for gel formation [1,2,3]. The aim of this study was to determine the effect of keeving on the chemical composition of fermented apple must and the volatile profile of apple brandies. We compared the application of keeving during spontaneous fermentation with fermentation carried out by *Saccharomyces cerevisiae* (SafSpirit HG-1). We also evaluated the effect of adding different doses of calcium chloride (0.1 g/L; 0.2 g/L; 0.4 g/L) on the selected parameters of fermented must (e.g., total extract, sugar-free extract, total acidity, free amino nitrogen, ethanol content, fermentation efficiency, and the profile of sugars) and distillates (concentration of volatile compounds and sensory analysis).

## 2. Materials and Methods

### 2.1. Fermentation

The apples used for fermentation were sourced from the Topaz cultivar, and harvested in Garlica Murowana (Małopolska district, Poland). Pectinesterase (Univar, Poland) was added to the crushed apples and left for 24 h. The crushed apples were then pressed using a Zottel hydropress (Zottel Trade d. o. o., Žalec, Slovenia) and divided into 2 L portions, which were placed in buckets with lids fitted with fermentation tubes. Calcium chloride (CaCl_2_) was added at varying concentrations (0.1 g/L, 0.2 g/L, and 0.4 g/L). The samples were either inoculated with a Saf-Spirit HG-1 yeast strain (Starowar, Warsaw, Poland) at 0.3 g d.w./L of must or allowed to ferment spontaneously. The fermentation took place at 20 °C for 30 days, during which time the brown cap gel was collected and fermentation continued (the timing of collection varied among the different samples). The musts that were not subjected to keeving served as the controls. The fermented musts were then collected and stored at −20 °C for chemical analysis, while the remaining must was distilled immediately.

### 2.2. Distillation

Samples after fermentation were distilled until the concentration of ethanol in the collected distillates was less than 0.5% (*w*/*v*) [5] and the final ethanol concentrations ranged from 9.9 to 18.8% (*v*/*v*) ethanol. The samples obtained were distilled again using a glass laboratory set with a Raschig column (40 cm) filled to 60% with rings (outer diameter, 8 mm; wall thickness, 1 mm; length, 20 mm) and a Liebieg cooler. During this distillation, 3 fractions were collected: the heads (2% of the distillate), the heart fraction (83%), and the tails (15%). The final ethanol concentrations by densitometry were approximately 65% (*v*/*v*) in the heart fraction, 80% (*v*/*v*) in the head fraction, and 20% (*v*/*v*) in the tail fraction. To prevent the loss of volatile compounds, the distillates were kept at 4 °C in sealed flasks until further analysis. This study reports only the results for the heart fractions.

### 2.3. Determination of Total Extract, Sugar-Free Extract, Titratable Acidity, Free Amino Nitrogen (FAN), and Ethyl Alcohol Content

The ethanol concentration, total extract, and sugar-free extract were assessed using the methods officially endorsed by the International Organisation of Vine and Wine [6]. The titratable acidity (TA) was measured with a Mettler DL 25 titrator (Mettler Toledo, Greifensee, Switzerland) and calculated based on the volume of 0.1 M NaOH used for titration, with the results expressed as grams of malic acid per litre. Free amino nitrogen (FAN) was quantified using the ninhydrin method. A 1 mL sample of fresh apple must or fermented must was diluted to 50 mL with distilled water, and 2 mL of this solution was placed in 16 mm × 150 mm test tubes. Ninhydrin colour reagent (1 mL) was added, and the tubes were heated in a boiling water bath for 16 min. After heating, the tubes were cooled in a cold-water bath, 5 mL of diluent reagent was added, mixed, and the absorbance was recorded at 575 nm against a blank containing 200 μL of water instead of the sample. A standard solution of 2 mL diluted glycine (10.72 mg/L) was used for the calibration [7]. The fermentation efficiency (%) was determined by comparing the ratio of sugar loss during fermentation to the amount of ethyl alcohol produced, based on fermentation stoichiometry (0.511 g of ethyl alcohol is produced from 1 g of reducing sugars, or 0.538 g from 1 g of sucrose).

### 2.4. Quantification of Sugar Content by High-Performance Liquid Chromatography (HPLC)

The sugar profile was analysed using high-performance liquid chromatography with a Shimadzu (Kyoto, Japan) NEXERA XR system, equipped with an RF-20A refractometer detector, following the method described by Januszek et al. (2020) [5].

The samples collected before and after fermentation were centrifuged (MPW-65R, MPW Med. Instruments, Warszawa, Poland) at 14,000× *g* for 5 min, and the fresh must was diluted with water. Separation was carried out on an Asahipak NH2P-50, 4.6 mm × 250 mm Shodex column (Showa Denko America, Munich, Germany), maintained at 30 °C. An aqueous solution of 70% acetonitrile was used as the mobile phase, and isocratic elution at a flow rate of 0.8 mL/min was performed for 16 min.

### 2.5. Analysis of Volatile Compounds Using GC-FID (Gas Chromatography with Flame Ionization Detection) and SPME-GC-MS (Solid Phase Microextraction Coupled with Gas Chromatography-Mass Spectrometry)

Selected volatile compounds were analysed using gas chromatography-flame ionization detection (GC-FID) as detailed by Januszek et al. [8]. The analysis was conducted on a Hewlett Packard 5890 Series II chromatograph system following previously established methods. The volatile concentrations were recalculated and reported as mg/L 100% (*v*/*v*).

For the SPME-GC-MS method, 2 mL of saturated saline containing an internal standard solution (5 mg/L of 4-methyl-2-pentanol and 0.05 mg/L of ethyl nonanoate, Sigma-Aldrich, Saint Louis, MO, USA) and 0.05 mL of ethanol were added to a 10 mL vial. The SPME device (Supelco Inc., Bellefonte, PA, USA) coated with a 100 μm polydimethylsiloxane fibre was conditioned by placing it in the GC injector port at 250 °C for 1 h. For sampling, the fibre was exposed to the headspace, with stirring (300 rpm) at 60 °C for 30 min. The SPME device was then inserted into the injector port of the Agilent Technologies 7890B chromatograph system (Agilent Technologies, Santa Clara, CA, USA), equipped with a LECO Pegasus HT High Throughput TOFMS (time-of-flight mass spectrometry), and held in the inlet for 3 min. The SPME process was automated using the GERSTEL MultiPurpose Sampler (MPS) (GERSTEL GmbH, Mülheim, Germany).

Separation was achieved using an Rtx-1ms capillary column (Crossbond 100% dimethyl polysiloxane, 30 m × 0.53 mm × 0.5 μm). The detector temperature was set to 250 °C, and the column was heated according to the following program: 40 °C for 3 min, then increased at a rate of 8 °C/min to 230 °C, and held at this temperature for 9 min. Helium was used as the carrier gas at a constant flow rate of 1.0 mL/min. Electron impact ionization was performed at 70 eV; the ion source and connecting parts were maintained at 250 °C. An analyte transfer was performed in splitless mode with the mass spectrometer detector set to scan mode from *m*/*z* = 40 to *m*/*z* = 400. The volatiles were identified using the mass spectral libraries and linear retention indices derived from a series of C6 to C30 n-alkanes. The quantity of components was determined semi-quantitatively by measuring the relative peak area of each identified compound, using the NIST 20 database and comparing it to the internal standard (ethyl nonanoate for esters, 4-methyl-2-pentanol for other compounds).

### 2.6. Sensory Analysis

The sensory evaluation of the brandies involved assessing eight aroma descriptors (fruity, citrus, floral, grassy, sweet, smoky, pungent, yeasty) on a 5-point hedonic scale using a Quantitative Descriptive Analysis (QDA). Ten panellists, selected from the scientific staff of the Faculty of Food Technology and Human Nutrition, were trained extensively in sensory analysis as a part of their curriculum. An aroma assessment was conducted using a set of standards provided to the panellists beforehand [9]. Panellists were first required to identify the standards to ensure their proficiency. Only those who successfully passed this identification course were included in the sensory analysis.

Prior to an evaluation, the apple brandies were diluted to 40% vol EtOH. The samples were coded and presented to the panellists in a randomized order.

### 2.7. Statistical Analysis

All analyses were conducted in quintuplicate, with results expressed as arithmetic means ± standard deviation. A statistical analysis was carried out using R 3.5.0 (Vienna, Austria). A MANOVA was executed using the lm function for linear models, and the Tukey’s test was applied via the honest significant difference (HSD) test function from the ‘agricolae’ package. A MANOVA considered variables such as different concentrations of CaCl2 and the fermentation types (with *Saccharomyces cerevisiae* SafSpirit HG-1 yeast or spontaneous fermentation). The sensory analysis results were analysed using the one-way analysis of variance (ANOVA), followed by the Pearson’s correlation test for each aroma descriptor to assess its impact on the “overall score”. The principal component analysis (PCA) was made for the volatile compounds and oenological parameters using the SPSS version 23 software (Chicago, IL, USA).

## 3. Results and Discussion

### 3.1. Selected Chemical Parameters of Fresh and Fermented Apple Musts Obtained After Keeving

On the basis of the two-way analysis of variance, it can be concluded that the doses of calcium chloride added had a greater effect on the ethanol concentration, total extract, and fermentation efficiency than the different types of fermentation. A different trend was observed for the sugar-free extract and for the other parameters—both variables had the same significant effect.

The apples from which cider is made should have a higher concentration of tannins and sugars and an appropriate acidity, as a low pH could disrupt enzyme activity. The optimal acidity for cider is about 0.5% malic acid [10]. In the case of our results, the titratable acidity of the musts was slightly below the optimal acidity (more than 3.5 g of malic acid/L), but this did not affect the keeving process and a brown layer formed on the surface of all the samples during fermentation. The total acidity during fermentation was slightly higher than the apple must acidity in most samples. However, the total acidity of the fermented musts decreased with an increasing addition of CaCl_2_ (Table 1). Some studies have confirmed that the addition of CaCl_2_ to fruit musts reduces their acidity [11]. CaCl_2_ is widely used as an acidity regulator and stabiliser in fruit and vegetable preserves.

Fresh, ripe apples contain approximately 10–13% total sugars, of which fructose is the dominant sugar [12]. The apple cultivar Topaz used in the current study had a reasonable total extract (121 g/L) and a relatively high sugar content (112 g/L). Fructose dominated the sugar profile (63.51 g/L—over 55% of the total sugars), then glucose and sucrose were present at lower concentrations of 24.25 g/L and 24.36 g/L, respectively (Table 2). Jakopic et al. [13] showed that cultivar Topaz had the highest amount of sucrose (40 g/L), and the concentration of total sugars was 94.4 g/L. The differences could be related to the growing and weather conditions.

As a result of the use of defecation, fermentation is slower and stops spontaneously, leaving some unfermented sugar [14]. Therefore, the concentration of fructose in the fermented control samples was 0.4 g/L, whereas it was about 3 g/L in the musts fermented after keeving (Table 2). Glucose was not found in any of the fermented samples.

The typical ABV (alcohol by volume) in a European keeved brut cider is between 4% and 5%, with residual sugars around 28 g/L. This technique ensures the relative microbial stability of the unpasteurised cider despite the presence of residual sugars. The concentration of glycerol in cider ranges from 3 to 6 g/L [15] and the concentration of these compounds in the samples analysed was within this range (Table 2). The samples fermented after keeving had lower concentrations of glycerol, which could be due to lower sugar consumption by the yeast during fermentation after keeving. In addition, the amount and composition of the nitrogen source has a pronounced effect on glycerol formation, so decreasing the nutrient concentrations could decrease the glycerol concentration [16,17].

The preferred ethanol concentration in cider is strongly dependent on the production region, e.g., in the United States it should be 7% or less, while in France it is below 4% [10,18]. In the current study, the concentration of ethanol was higher in the control samples (6.3% vol., Table 1) than in the musts fermented after the defecation process (from 5.4% vol. to 5.9% vol.), which was dependent on the concentration of residual sugars in these samples. Therefore, the fermentation efficiency was higher in the non-defecated variants.

Another important factor determining the quality of fermented musts is nitrogen compounds, which serve as a nutrient for yeast [19]. For cider production, the concentration of nitrogen in must should be increased to 100 mg N/L. It is important to monitor the level of nitrogen compounds in the must as their excess can lead to undesirable flavours and microbial instability in alcoholic beverages [10,20,21,22]. According to the results presented by Gomis et al. [23], the concentration of nitrogen in apple cider was 97.8 mg/L. The concentration of free amino nitrogen in the musts analysed in the present study was much lower (51.2 mg/L, Table 1); therefore, 0.2 g/L (NH_4_)2HPO_4_ was added to the musts. Due to the involvement of nitrogen in gel formation, the nitrogen concentration in the fermented samples after keeving was about four times lower than that in the control samples.

**Figure 2:**
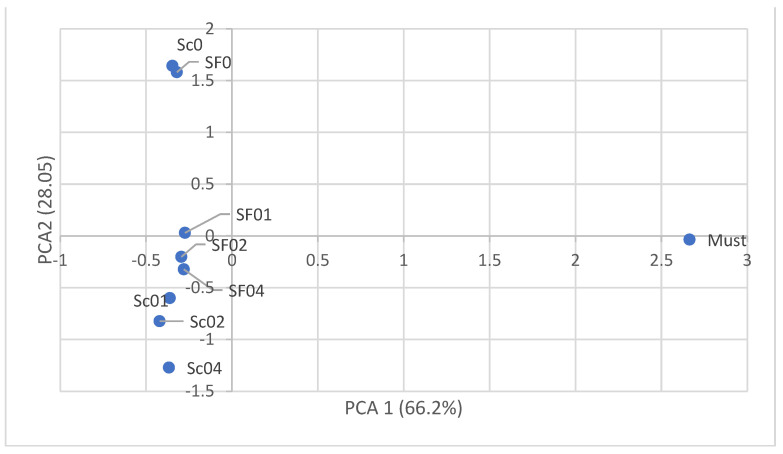
Principal component analysis showing the effect of different CaCl_2_ additions and types of yeast on the chemical composition of apple musts. SF—spontaneous fermentation; Sc—*Saccharomyces cerevisiae* SafSpirit HG-1. Doses of CaCl_2_: 0—0 g/L; 01—0.1 g/L; 02—0.2 g/L; and 04—0.4 g/L.

**Figure 3:**
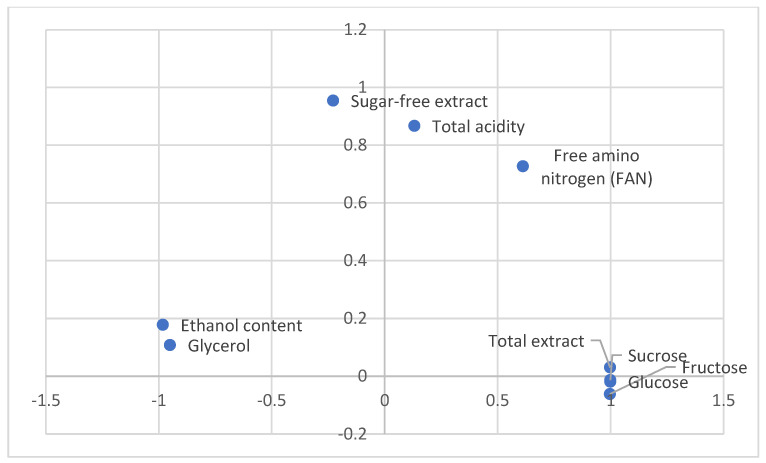
Principal component analysis for oenological parameters of fermented apple musts.

The PCA confirmed that the addition of calcium chloride had a greater effect on the oenological parameters, such as the ethanol concentration, total extract, and fermentation efficiency, than on the type of fermentation used (Figure 2). Moreover, the PCA confirmed that the total extract content is significantly influenced by the glucose and fructose levels (Figure 3). Similarly, the ethanol concentration is related to the glycerol content, and the sugar-free extract content depends on the total acidity and free amino nitrogen concentration.

### 3.2. Volatile Compounds in Apple Brandies Obtained from Musts After Keeving

The use of defecation, which involves the removal of nutrients from musts, could be linked to the transformation of volatile compounds. Branched-chain amino acids (Leu, Ile, Val) are converted into specific aldehydes with malty aromas, alcohols with fruity and alcoholic aromas, and acids with sweet, sour, rancid, rotten, fruity, and buttery aromas, depending on which amino acid, Leu, Ile, or Val, is catabolised. Aromatic amino acids (Phe, Tyr, Trp) are catabolized to compounds that contribute to flavours such as rose, flower, and bitter almond, as well as chemical, putrid, and faecal flavours [24]. Defecation increases the presence of reduced sulphides and other substances produced by yeast metabolism at low nitrogen concentrations. On the other hand, keeving is a typical practice in craft cider production and, if carried out correctly, can result in naturally sweet and sparkling ciders with an increased volatile content of fruity aroma [25]. Ledauphin et al. (2004) [26] analysed freshly distilled Calvados (a unique apple spirit produced in Normandy) and identified 93 volatile compounds specific to Calvados with compounds such as unsaturated alcohols, phenolic derivatives, and unsaturated aldehydes.

Esters were the most diverse group of volatiles analysed, consisting of almost 50 compounds (Table 3). Esters have fruity and floral aromas that are important for the sensory properties of alcoholic beverages [27]. In general, their concentration was closely related to the type of fermentation. Compared to the control, the concentration of some esters (e.g., n-ethyl propionate, ethyl lactate, ethyl tetradecanoate, ethyl pentadecanoate, methyl palmitate, ethyl palmitate, methyl linoleate, 2-phenylethyl hexanoate, isoamyl decanoate, isopropyl dodecanoate, hexyl decanoate, isoamyl laurate) was lower in the brandies obtained from the musts fermented spontaneously with the addition of CaCl_2_ and slightly higher in the samples fermented with *Saccharomyces cerevisiae* SafSpirit HG-1. When the amount of nutrients in the environment is limited and starvation occurs, microorganisms use the available nutrients for basic metabolic processes that allow them to survive and limit the formation of side metabolites such as volatiles. Many aromatic compounds are closely tied to nitrogen metabolism. For instance, the production of higher alcohols, as well as the fatty acids and esters associated with them, depends on the quality and quantity of the nitrogen sources. When nitrogen is limited, the production of higher alcohols increases through both the catabolic and anabolic biosynthetic pathways. Additionally, nitrogen metabolism influences other critical pathways, including sugar and sulphur metabolism, as well as the utilization of essential nutrients. This interplay can significantly impact the production of various flavour-active intermediates and end-products [28].

However, defecation increased the concentration of all the acetate esters (ethyl acetate, isobutyl acetate, butyl acetate, isoamyl acetate, hexyl acetate, octyl acetate, and phenylethyl acetate). Similar results were presented by Villière et al. [32], who stated that among the different clarification techniques (keeving, decanting, depectinisation), keeving had the most significant effect on the increase of acetates. Esters are formed during fermentation in the reaction between the alcohols produced by yeast and acyl-CoA. Slow fermentations or conditions that are stressful for the yeast result in a greater formation of higher alcohols, acetates, and fatty acid ethyl esters. In addition, it has been shown in wine that under slight nitrogen depletion, yeast synthesises more higher alcohols and more acetates can be obtained from these alcohols and acetyl-CoA [32].

The second most abundant group of volatile compounds in the apple brandies were alcohols (Table 3). Methanol is formed during the demethoxylation of esterified methoxyl groups in pectin [5]. Despite the addition of pectinoesterases during defecation, its concentration decreased slightly after fermentation. The lower methanol content in the brandies obtained from the samples fermented after keeving (Table 3) could be explained by the removal of the gel formed during the initial stage of fermentation, which contained significant amounts of pectin.

The amino acids present in apple must are precursors and intermediates in the biosynthesis of many volatile compounds. Higher alcohols are formed as a result of the catabolism of amino acids through the Erlich pathway [24,34,35]. According to the study by Santos et al. [33], the presence of aspartic acid, asparagine, glutamic acid, and alanine has a positive effect on the amount and diversity of the volatile compounds, especially higher alcohols (e.g., 2-phenylethanol and 3-methyl-1-buthanol). It can therefore be assumed that defecation (which reduces the nutrient content of the medium, including amino acids) could also reduce the amount of higher alcohols. On the other hand, it has been shown that in wines, yeasts synthesise more higher alcohols when the nitrogen compounds are slightly reduced [32]. In the study by Eleutério dos Santos et al. [34], aspartic acid, asparagine, and glutamic acid were the main compounds found in all nine apple musts.

According to our results, the concentration of most of the higher alcohols (isobutanol, 4-methyl-1-pentanol, 3-methyl-1-pentanol, 1-decanol, 1-dodecanol, and 1-tetradecanol) decreased after spontaneous fermentation and increased during fermentation with *Saccharomyces cerevisiae* SafSpirit HG-1. Regardless of the type of fermentation, the concentration of all other higher alcohols (butanol, 2-heptanol, 1-heptanol, 6-methyl-5-hepten-2-one, 2-ethyl-hexanol, 1-octanol, phenylethanol) and amyl alcohols increased. Thus, the increase in the synthesis of higher alcohols could be a result of low assimilable nitrogen, as more carboxylic acids (a-cetoacids) were available for the production of higher alcohols than for the synthesis of amino acids [36].

Similar dependencies were found for aldehydes and ketones. The concentration of acetaldehyde, dodecanal, and geranylaceton decreased after spontaneous fermentation and increased during the fermentation carried out with *Saccharomyces cerevisiae* SafSpirit HG-1, but a different phenomenon was observed in the case of nonanal. This tendency could be related to the concentrations of diethyl acetals (1,1-diethoxyethane, 1,1-diethoxy-propane, 1,1-diethoxybutane, and 1,1-diethoxypentane), which increased in the spontaneously fermented musts and decreased in the musts fermented with *Saccharomyces cerevisiae* SafSpirit HG-1. Acetals are common in spirituous beverages, especially in cognac, and are formed by the condensation of aldehydes with ethanol, probably during the distillation process. These compounds have a pleasant fruity, floral, and alcoholic aroma and their olfactory detection threshold is around 500 µg/L [31]. After keeving, the concentration of decanal and 2-furaldehyde diethyl increased slightly in all the analysed apple brandies.

Terpenes mainly come directly from fruits, but most terpenes are bound to sugar moieties in a must and could be released by the acids or glycosidase in fruits and yeast, ultimately leading to an increase in the terpene concentrations in a cider [35]. Keeving caused an increase in the terpene concentrations, with the exception of guaiacol. A slight decrease in the concentration of this compound may be related to its transformation into other compounds. Guaiacol is a precursor to several flavours such as eugenol and vanillin [37]. The concentration of the other 20 terpenes increased in the samples fermented after keeving with *Saccharomyces cerevisiae* SafSpirit HG-1 yeast, but the concentration of some terpenes (geranylacetone, β-damascenone, methyleugenol, (*E*)-β-famesene, (*Z*,*E*)-α-farnesene, β-ionone, α-farnesene, 2,3-dihydrofarnesol, farnesol, and nerolidol) decreased in the spontaneously fermented must. The increased concentration of terpenes after defecation may be related to the fact that clarification increases glycosidase activity, leading to a greater release of volatiles from the odourless glycosidic structures [32].

**Figure 4:**
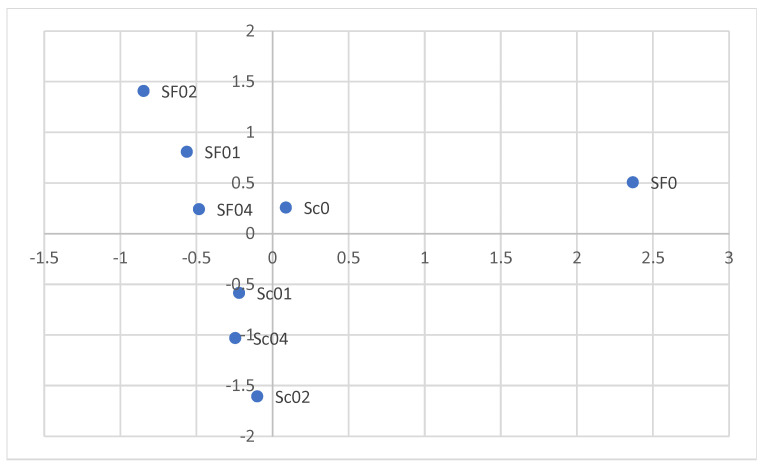
Principal component analysis showing the effect of different CaCl_2_ additions and types of yeast on the volatile compounds present in apple spirits. SF—spontaneous fermentation; Sc—*Saccharomyces cerevisiae* SafSpirit HG-1. Doses of CaCl_2:_ 0—0 g/L; 01—0.1 g/L; 02—0.2 g/L; 04—0.4 g/L.

**Figure 5:**
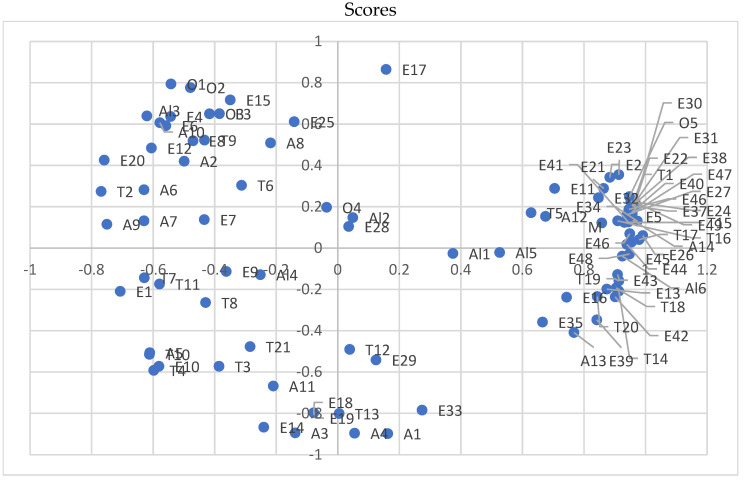
Principal component analysis showing the effect of different CaCl_2_ additions and types of yeast on the volatile compounds present in apple spirits. Esters (E); Methanol (M); Higher Alcohols (A); Aldehydes and ketones (Al), Terpenoids (T); Other compounds (O). The reference of the compound name to the symbol is summarized in Table 3.

The concentration of CaCl_2_ and type of yeast had significant effect on the qualitative and quantitative profiles of the volatile compounds. The PCA showed that the use of spontaneous fermentation was associated with higher ester concentrations in the apple spirits analysed. On the other hand, samples fermented with *Saccharomyces cerevisiae* SafSpirit HG-1 yeast showed a higher content of terpenes and higher alcohols (Figure 4). The use of keeving also had a significant effect on the formation of volatile compounds. The spirits produced after keeving were found to have a higher content of terpenes as well as higher alcohols and some esters, such as ethyl butyrate, ethyl crotonate, methyl octanoate, and others (Figure 5).

### 3.3. Sensory Analysis

**Figure 6:**
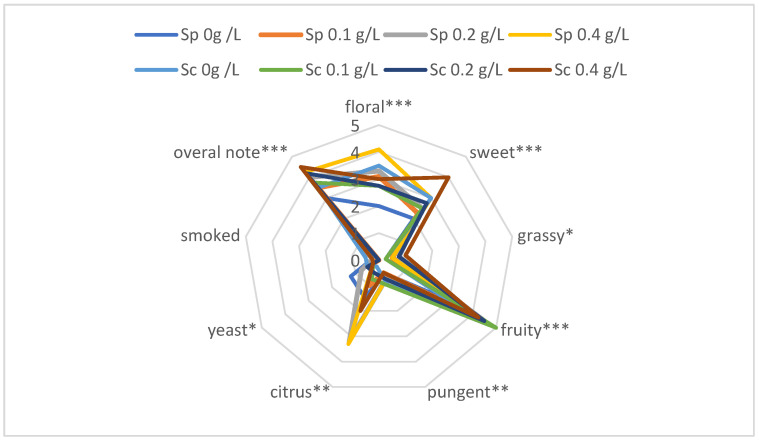
Characteristic aroma features of apple brandies obtained from musts fermented with the addition of CaCl_2_; n = 5, STD < 5%. Sp—spontaneous fermentation; Sc—*Saccharomyces cerevisiae* SafSpirit HG-1 (0; 0.1; 0.2; 0.4 g/L—doses of CaCl_2_ added); *, **, and ***—the significance at 0.05, 0.01, and 0.005 by least significant difference, respectively.

All the analysed apple brandies were described as clear and received maximum scores for this descriptor. The majority of the samples fermented with *Saccharomyces cerevisiae* SafSpirit HG-1 obtained high scores for the parameter “overall note” (overall acceptability of the samples tested) and all of them obtained higher scores than the control samples (Figure 6). Most of the samples fermented after defecation received high scores for the floral and fruity parameters (from 3.0 to 4.5 points, Figure 6). The intense floral, fruity, and sweet aromas could be attributed to the higher concentration (compared to the control samples) of some esters, e.g., ethyl acetate, isobutyl acetate, butyl acetate, isoamyl acetate, hexyl acetate, and phenylethyl acetate. These compounds have intense floral and fruity aromas, e.g., ethyl acetate has a pleasant, ethereal, fruity, brandy-like aroma reminiscent of pineapple, which is slightly nauseating at high concentrations and its aroma threshold is between 5 ppb and 5 ppm. The concentration of ethyl acetate in apple distillates varies and significantly impacts flavour development. At excessively high levels, it can impart undesirable glue-like notes onto the distillates. For the registered designation of origin ‘Calvados Domfrontais’, the maximum allowable concentration of ethyl acetate is 350 g per hectolitre of pure alcohol [29]. In the samples analysed, both those obtained by defecation and the controls, the ethyl acetate concentrations were significantly lower, around 140 mg/L. Similar concentrations have been reported by Dimitrov and Ivanova [30], who found 170 mg/L for grape brandy and 155 mg/L for plum brandy. Slightly higher concentrations were observed by Cortés et al. [38], with around 400 mg/L for grape brandy. Further compounds imparting fruity aromas were isobutyl acetate, which has a fruity (currant–pear), floral (hyacinth–rose) odour and a characteristic ether-like, slightly bitter taste, with an odour threshold ranging from 65 to 880 ppb, and butyl acetate, which has a strong, fruity aroma, and a burning and then sweet taste reminiscent of pineapple [37]. Le Quéré et al. [25] claimed that defecation increased the fruity aroma in alcoholic beverages. The herbaceous aroma detected in all the samples obtained after defecation could be associated with the highest concentration of hexanol and linalool oxide, resulting in earthy, floral, herbaceous, and lavender odours, and its flavour threshold is 5 ppm [37]—this value was exceeded in the samples analysed. The concentration of hexanol in the samples ranged from 26.1 mg/L to 86.8 mg/L. Madrera and Valles [39] reported similar concentrations, ranging from 35.5 mg/L to 61.3 mg/L, in cider spirits. They also noted that higher hexanol concentrations were associated with the use of fresh fruit. In contrast, when apple concentrate was used, the hexanol content was lower, while the furfural content increased, significantly affecting the sensory characteristics of the distillates. The concentration of linalool oxide in our spirits averaged 0.7 mg/L. Similar results were obtained by Dimitrov and Ivanova [30] in grape and plum spirits (average 0.3 mg/L). Moreover, we obtained similar concentrations of volatile compounds in apple spirits in our previous studies [5,8]. All the apple brandies scored relatively high for the citrus parameter. This could be related to higher concentrations of the myrcene, limonene, and citral compounds, which are characteristic of a citrus aroma. The concentration of these compounds was higher in the spontaneously fermented samples, so these brandies received higher scores for this parameter compared to the variants fermented with *Saccharomyces cerevisiae* SafSpirit HG-1 (Figure 6).

The brandies produced from the musts fermented with *Saccharomyces cerevisiae* SafSpirit HG-1 after defecation obtained the highest average scores for “overall note” (more than 4.0 pt), regardless of the concentration of CaCl_2_ added. This means that this method, carried out with a properly selected yeast strain, could be feasible for the distilling industry. The Pearson test showed positive and negative correlations between some descriptors (floral, sweet, fruity, or citrus—positive; pungent, yeasty—negative) and the overall note. Properly carried out keeving has a positive effect on the sensory characteristics of apple brandy, enhancing its fruity aroma.

The results obtained indicate that the keeving method, carried out with a properly selected yeast strain, could be feasible for the distilling industry. Using this method, we can obtain spirits with a more intense floral and fruity aroma. In addition, we can reduce the concentration of components that affect its quality.

## 4. Conclusions

The two-way analysis of variance revealed that the addition of calcium chloride (CaCl_2_) had a more substantial impact on the ethanol concentration, total extract, and fermentation efficiency than the type of fermentation. Conversely, for the sugar-free extract and other parameters, both CaCl_2_ and the fermentation type had similar significant effects. This underscores the critical role of CaCl_2_ in influencing key chemical parameters during fermentation. Keeving resulted in slower fermentation with some residual sugars remaining, as evidenced by the higher fructose concentration in the fermented musts post-keeving. This technique contributed to a lower ethanol concentration compared to non-keeved samples, likely due to reduced fermentation efficiency. The concentration of glycerol, a byproduct of fermentation, was lower in the keeved samples, potentially due to the decreased sugar consumption by yeast and the lower nitrogen levels. This study highlighted that the nitrogen concentration in the must was significantly lower in the fermented samples post-keeving compared to the controls. This lower nitrogen level impacted the formation of volatile compounds, particularly higher alcohols and esters. The findings suggest that adequate nitrogen levels are crucial for optimal yeast performance and volatile compound production.

Keeving and the type of fermentation influenced the volatile compound profile significantly. Keeving enhanced the terpene concentrations and affected the presence of higher alcohols and esters. Spontaneous fermentation generally led to higher concentrations of certain esters, while fermentation with *Saccharomyces cerevisiae* SafSpirit HG-1 yeast resulted in elevated levels of terpenes and higher alcohols.

The sensory analysis revealed that the apple brandies fermented with *Saccharomyces cerevisiae* SafSpirit HG-1 yeast scored higher for overall acceptability compared to the controls. The brandies produced after defecation generally received high scores for floral and fruity aromas. The increased concentration of esters, such as ethyl acetate and isobutyl acetate, contributed to these desirable sensory attributes. Additionally, the brandies from the spontaneous fermentation were noted for their citrus aromas due to higher levels of myrcene, limonene, and citral.

This study underscores the importance of both the CaCl_2_ addition and fermentation techniques in shaping the chemical and sensory profiles of apple brandies. Keeving, while slowing down fermentation and affecting ethanol yields, enhances certain volatile compounds and aromas. The fermentation type and practices such as defecation have a profound impact on the final product’s quality, influencing both the chemical composition and sensory characteristics.

## Figures and Tables

**Table 1:** The impact of different types of fermentation and doses of CaCl_2_ used in the keeving process on the oenological parameters of apple musts and fermented apple musts.

	Oenological Parameters	Before Fermentation Process					
Apple Cultivar		Total Extract	Sugar-Free Extract	Total Acidity	Free Amino Nitrogen (FAN)		
		[g/L]			[mg/L]		
Topaz		121.0 ± 0.9	9.2 ± 0.3	3.58 ± 0.05	51.22 ± 1.3
	**Oenological Parameters**	**After Fermentation Processes**			
**Doses of** **CaCl_2_ [g/L]**		**Total Extract**	**Sugar-Free Extract**	**Total Acidity**	**Free Amino Nitrogen (FAN)**	**Ethanol Content**	**Fermentation Efficiency**
		**[g/L]**			**[mg/L]**	**[% vol.]**	**[%]**
Spontaneous fermentation	0 g/L	16.0 a ± 0.3	15.4 a ± 0.5	3.87 a ± 0.15	46.8 a ± 1.25	6.3 a ± 0.3	83.2 a ± 4.0
	0.1 g/L	13.1 b ± 0.1	10.6 b ± 0.4	3.66 ab ± 0.17	18.06 c ± 1.89	5.6 ab ± 0.3	74.4 ab ± 4.2
	0.2 g/L	12.4 bc ± 0.4	10.1 bc ± 0.4	3.64 ab ± 0.77	11.13 d ± 0.88	5.9 ab ± 0.2	78.8 ab ± 2.0
	0.4 g/L	11.9 c ± 0.5	9.4 cde ± 0.4	3.59 a ± 0.35	12.91 d ± 1.99	5.4 b ± 0.2	71.8 b ± 2.0
*Saccharomyces cerevisiae* SafSpirit HG-1	0 g/L	16.4 a ± 0.2	15.5 a ± 0.5	4.13 a ± 0.25	39.53 b ± 1.23	6.3 a ± 0.2	83.2 a ± 2.6
	0.1 g/L	12.9 b ± 0.3	9.9 bcd ± 0.3	3.04 bc ± 0.28	14.04 d ± 0.77	5.5 b ± 0.4	72.7 b ± 4.8
	0.2 g/L	12.3 bc ± 0.2	9.0 de ± 0.3	2.97 c ± 0.10	11.68 d ± 0.77	5.6 ab ± 0.3	73.6 ab ± 4.2
	0.4 g/L	12.1 c ± 0.2	8.7 e ± 0.3	2.41 c ± 0.24	13.4 d ± 1.82	5.4 b ± 0.2	71.3 b ± 2.6
Significance	type of fermentation	ns	**	**	**	ns	ns
	doses of CaCl_2_	***	***	**	**	***	***

Same letters next to mean values within columns indicate the lack of statistically significant differences at *p* < 0.05; n = 3; ns—not significant; 0.001 = ***; 0.01 = **.

**Table 2:** The impact of different types of fermentation and doses of CaCl_2_ used in the keeving process on the profile of sugars in apple musts and fermented apple musts.

	Sugars	Before Fermentation Process			
Apple Cultivars		Glycerol	Fructose	Glucose	Sucrose
		[g/L]			
Topaz		0.00 ± 0.00	63.51 ± 1.40	24.25 ± 1.33	24.36 ± 0.44
	**Sugars**	**After Fermentation Processes**			
**Doses of** **CaCl_2_ [g/L]**		**Glycerol**	**Fructose**	**Glucose**	**Sucrose**
		**[g/L]**			
Spontaneous fermentation	0 g/L	5.72 a ± 0.09	0.43 b ± 0.02	0.00 ± 0.00	0.15 a ± 0.01
	0.1 g/L	4.51 cd ± 0.27	2.22 a ± 0.05	0.00 ± 0.00	0.23 a ± 0.06
	0.2 g/L	4.01 d ± 0.33	2.01 a ± 0.07	0.00 ± 0.00	0.26 a ± 0.02
	0.4 g/L	4.54 bcd ± 0.19	2.30 a ± 0.12	0.00 ± 0.00	0.28 a ± 0.06
*Saccharomyces cerevisiae* SafSpirit HG-1	0 g/L	5.63 ab ± 0.19	0.38 b ± 0.01	0.00 ± 0.00	0.15 a ± 0.01
	0.1 g/L	5.24 abc ± 0.74	2.81 a ± 1.00	0.00 ± 0.00	0.23 a ± 0.02
	0.2 g/L	5.82 a ± 0.24	3.09 a ± 0.23	0.00 ± 0.00	0.29 a ± 0.12
	0.4 g/L	5.24 abc ± 0.60	3.04 a ± 0.48	0.00 ± 0.00	0.27 a ± 0.08
Significance	type of fermentation	**	**	ns	ns
	doses of CaCl_2_	*	***	ns	**

Same letters next to the mean values within columns indicate the lack of statistically significant differences at *p* < 0.05; n = 3; ns—not significant; 0.001 = ***; 0.01 = **; 0.05 = *.

**Table 3:** The influence of different doses of CaCl_2_ used in the keeving process on the concentration of volatile compounds in apple brandies [mg/L 100°].

	Volatile Compounds *	LRI ^1^	Spontaneous Fermentation				*Saccharomyces cerevisiae* SafSpirit HG-1				Significance	Method	Olfactory Detection Threshold	Odour Description **
			Control Sample	Doses of CaCl_2_ [g/L]			Control Sample	Doses of CaCl_2_ [g/L]						
				0.1	0.2	0.4		0.1	0.2	0.4				
Symbol for PCA	Esters													
E1	Ethyl acetate	614	107.12	140.43	139.23	137.23	102.09	137.8	139.21	138.02	**	GC-MS GC-FID	5000	dry fruity, musty, pineapple
E2	n-Ethyl propionate	678	91.67	19.94	14.93	9.86	2.09	2.63	3.21	7.63	***	GC-MS	0.007	pineapple
E3	Isobutyl acetate	763	290.1	582.3	462.3	413.2	532.2	232.2	286.4	320.3	***	GC-MS GC-FID	100	mild, fruity
E4	Ethyl butyrate	789	1.28	5.18	9.71	2.92	2.41	1.83	1.08	4.19	***	GC-MS	0.015	fruity–pineapple, banana
E5	Ethyl lactate	797	16.59	1.89	0.77	2.17	1.16	3.96	1.44	1.49	***	GC-MS	0.2	mild ethereal–buttery
E6	Butyl acetate	799	0.11	0.47	0.74	0.44	0.04	0.10	0.19	0.23	***	GC-MS	0.63	fruity
E7	Ethyl (*E*)-crotonate	844	0.00	0.00	0.54	0.00	0.17	0.14	0.16	0.28	***	GC-MS	4	sour, caramellic–fruity
E8	Ethyl 2-methylbutyrate	841	0.07	0.19	0.47	0.08	0.09	0.09	0.08	0.21	***	GC-MS	0.006	green–fruity pungent
E9	Ethyl 3-methylbutyrate	843	0.00	0.00	0.14	0.00	0.00	0.00	0.07	0.13	***	GC-MS	0.015	fruity
E10	Isoamyl acetate	876	138.32	143.92	149.03	152.12	139.03	155.44	154.02	153.11	***	GC-MS GC-FID	0.22	banana, pear
E11	Ethyl hexanoate	986	3.11	0.72	2.26	1.88	2.12	1.69	1.44	1.59	***	GC-MS GC-FID	10	sweet, waxy, fruity, apple, grape, oily, brandy
E12	Hexyl acetate	1006	0.23	2.14	2.39	0.79	0.23	0.52	0.68	1.21	***	GC-MS	0.012	green fruity, sweet
E13	Isoamyl lactate	1065	3.05	0.00	0.00	0.00	2.11	0.59	1.58	0.76	***	GC-MS	0.89	fruity, creamy, nutty
E14	Methyl octanoate	1108	0.10	0.11	0.12	0.12	0.15	0.18	0.23	0.16	***	GC-MS	na	waxy
E15	Ethyl benzoate	1142	0.17	0.38	0.44	0.20	0.27	0.39	0.06	0.06	***	GC-MS	0.6	fruity, dry, musty
E16	Diethyl succinate	1149	6.11	3.59	2.69	2.14	5.55	4.59	4.96	3.32	*	GC-MS GC-FID	10	faint, pleasant
E17	Ethyl octanoate	1180	11.48	11.43	11.33	11.06	6.23	5.58	4.44	3.12	***	GC-MS GC-FID	10	fruity, floral
E18	Octyl acetate	1196	0.00	0.00	0.00	0.00	0.00	0.00	0.05	0.02	***	GC-MS	6	fruity, slightly waxy floral
E19	Ethyl phenylacetate	1210	0.00	0.00	0.00	0.00	0.00	0.00	0.05	0.02	***	GC-MS	140	sweet, honey, rose, fruity
E20	phenylethyl acetate	1228	58.53	71.33	76.78	72.22	60.32	66.04	65.32	64.43	***	GC-MS GC-FID	na	floral
E21	Methyl decanoate	1330	0.17	0.01	0.02	0.00	0.11	0.04	0.00	0.00	***	GC-MS	-	fermented
E22	Ethylphenyl propionate	1339	0.19	0.00	0.00	0.00	0.01	0.00	0.00	0.00	***	GC-MS	-	sweet, fruity, honey-like
E23	Isobutyl octanoate	1351	0.06	0.01	0.01	0.00	0.01	0.01	0.00	0.00	***	GC-MS	1.1	fruity
E24	α-Phenylethyl butyrate	1431	0.12	0.01	0.01	0.00	0.01	0.01	0.01	0.00	***	GC-MS	0.015	floral. musty sweet floral yeasty strawberry
E25	Isopentyl octanoate	1445	0.47	0.02	002	0.04	0.34	0.24	0.07	0.07	***	GC-MS	2.5	sweet, oily
E26	2-Methylbutyl octanoate	1449	0.18	0.00	0.00	0.00	0.04	0.03	0.03	0.01	***	GC-MS	na	sweet, oily, fruity, green, soapy, pineapple, coconut
E27	Phenethyl 2-methylbutyrate	1466	0.03	0.00	0.00	0.00	0.01	0.01	0.00	0.00	***	GC-MS	na	sweet floral fruity
E28	Propyl decanoate	1472	0.00	0.00	0.00	0.00	0.01	0.00	0.00	0.00	***	GC-MS	na	waxy
E29	Ethyl 9-decenoate	1366	0.18	0.01	0.01	0.03	0.05	0.66	0.24	0.18	***	GC-MS	-	fruity, fatty
E30	Methyl laurate	1507	0.32	0.01	0.01	0.01	0.02	0.02	0.01	0.01	***	GC-MS	na	fatty, floral
E31	Isobutyl decanoate	1546	0.40	0.00	0.01	0.00	0.04	0.02	0.00	0.00	***	GC-MS	na	fermented
E32	Hexyl octanoate	1565	0.04	0.00	0.00	0.00	0.03	0.00	0.00	0.00	***	GC-MS	na	vegetable, fruity
E33	2-phenylethyl hexanoate	1611	0.21	0.00	0.06	0.0	0.01	0.12	0.40	0.33	***	GC-MS	0.0025	sweet, honey, floral, waxy, woody, green, banana, pineapple
E34	Isoamyl decanoate	1641	3.69	0.05	0.04	0.06	0.25	0.47	0.24	0.24	***	GC-MS	na	waxy
E35	2-Methylbutyl valerate	1650	0.14	0.01	0.01	0.02	0.01	0.03	0.05	0.14	***	GC-MS	20	apple, green
E36	Isopropyl dodecanoate	1617	0.39	0.01	0.01	0.04	0.04	0.03	0.22	0.20	***	GC-MS	na	na
E37	Propyl dodecanoate	1694	0.09	0.00	0.00	0.00	0.00	0.00	0.00	0.00	***	GC-MS	26	alcohol
E38	Methyl tetradecanoate	1707	0.09	0.00	0.00	0.00	0.00	0.00	0.00	0.00	***	GC-MS	na	orris-like
E39	Isobutyl laurate	1753	0.07	0.00	0.00	0.01	0.02	0.02	0.00	0.00	***	GC-MS	na	waxy
E40	Hexyl decanoate	1784	0.65	0.00	0.00	0.07	0.01	0.05	0.03	0.00	***	GC-MS	0.5	fresh, green
E41	Ethyl tetradecanoate	1790	14.55	0.18	0.49	0.36	0.30	3.70	0.85	0.44	***	GC-MS	na	sweet, waxy, violet
E42	2-phenylethyl octanoate	1820	1.66	0.01	0.03	0.04	0.04	0.18	0.77	0.51	***	GC-MS		sweet, floral, rose-like
E43	Isoamyl laurate	1844	0.21	0.01	0.01	0.03	0.01	0.01	0.07	0.08	***	GC-MS	400	wine, waxy, oily
E44	Ethyl pentadecanoate	1880	0.82	0.02	0.02	0.04	0.01	0.26	0.12	0.09	***	GC-MS	na	sweet
E45	Methyl palmitate	1927	0.26	0.01	0.02	0.03	0.01	0.04	0.06	0.04	***	GC-MS	na	fatty, oily
E46	Ethyl *E*-11-hexadecenoate	1974	3.69	0.20	0.19	0.35	0.14	0.19	0.52	0.86	***	GC-MS	-	-
E47	Ethyl palmitate	1990	38.79	1.39	0.94	1.29	0.30	2.81	2.21	1.69	***	GC-MS	18	waxy
E48	Methyl linoleate	2171	0.76	0.06	0.02	0.06	0.02	0.05	0.21	0.16	***	GC-MS	3.2	fatty, waxy
E49	Ethyl stearate	2189	0.22	0.01	0.00	0.00	0.01	0.00	0.00	0.00	***	GC-MS	na	waxy
	Methanol													
M1	Methanol	361	915.2	702.4	743.2	687.2	840.3	709.3	772.8	714.6	***	GC-MS GC-FID	4.2–5960	similar to ethanol
	Higher alcohols													
A1	Isobutanol	617	182.3	113.4	93.4	132.3	223.6	273.4	274.6	282.5	***	GC-MS GC-FID	0.66	sweet, musty
A2	Butanol	658	66.1	77.3	97.7	81.2	60.2	91.3	66.4	67.2	***	GC-MS GC-FID	20	sweet, alcohol
A3	4-Methyl-1-pentanol	838	0.00	0.00	0.00	0.00	72.2	111.3	132.2	103.2	***	GC-MS	na	nutty
A4	3-Methyl-1-pentanol	850	27.3	0.00	0.00	0.00	52.2	93.2	96.2	91.3	***	GC-MS	10	wine-like, cocoa
A5	Hexanol	865	26.1	71.1	57.4	61.2	24.3	73.4	86.8	67.3	***	GC-MS GC-FID	0.01	herbal
A6	2-Heptanol	900	0.16	0.44	0.92	0.62	0.19	0.75	0.42	0.27	***	GC-MS	na	citrus, herbal
A7	1-Heptanol	954	0.19	0.49	0.91	0.39	0.19	0.68	0.55	0.40	***	GC-MS	0.49	green
A8	6-methyl-5-Hepten-2-one	967	0.11	0.14	0.16	0.16	0.07	0.03	0.10	0.11	***	GC-MS	50	citrus, green
A9	2-ethyl-hexanol	1017	1.18	6.75	10.91	6.04	1.19	6.28	6.14	6.87	***	GC-MS	na	floral–rosy
A10	1-Octanol	1070	0.63	1.29	1.94	0.93	0.70	1.08	0.79	0.73	***	GC-MS	110–130	pungent
A11	2-phenylethanol	1084	0.38	4.17	1.91	0.51	14.67	10.98	11.25	13.34	***	GC-MS	1000	sweet, floral, rose-like
A12	1-Decanol	1272	0.36	0.28	0.05	0.05	0.02	0.10	0.14	0.08	***	GC-MS	0.00077	sweet, fat-like
A13	1-Dodecanol	1460	0.64	0.15	0.06	0.12	0.02	0.27	0.49	0.23	***	GC-MS	7.1	floral
A14	1-Tetradecanol	1664	1.13	0.02	0.01	0.02	0.01	0.03	0.05	0.14	***	GC-MS	na	waxy
	Aldehydes and ketones													
Al1	Acetaldehyde	412	170.2	113.4	162.3	104.5	142.4	164.4	122.3	175.4	***	GC-MS GC-FID	15–120	ethereal
Al2	2-Furaldehyde diethyl acetal	1078	0.28	0.25	0.31	0.25	0.19	0.25	0.28	0.25	*	GC-MS	na	fruity
Al3	Nonanal	1083	0.19	0.81	0.75	0.38	0.53	0.52	0.25	0.30	***	GC-MS	1	rose–orange
Al4	Decanal	1182	0.12	0.19	0.20	0.08	0.00	0.14	0.20	0.17	***	GC-MS	0.3–0.4	fruity, floral
Al5	Dodecanal	1401	0.06	0.06	0.01	0.01	0.01	0.02	0.03	0.04	***	GC-MS	2	violet, pine
Al6	Geranylaceton	1443	1.02	0.07	0.05	0.13	0.02	0.01	0.33	0.23	***	GC-MS	100	sweet, fruity-floral
Terpenoids														
T1	Farnesal	1730	0.15	0.00	0.00	0.00	0.00	0.00	0.00	0.00	***	GC-MS	0.01	minty, floral
T2	Limonene	1020	0.35	0.48	0.59	0.49	0.39	0.41	0.46	0.48	**	GC-MS GC-FID	65	lemon and citrus
T3	Linalool oxide	1078	0.57	0.55	0.69	0.91	0.57	0.73	0.82	0.88	***	GC-MS GC-FID	5	sweet, earthy, floral, spice, lavender
T4	Linalool	1094	0.14	0.27	0.23	0.21	0.22	0.35	0.31	0.28	***	GC-MS GC-FID	6	flower and lavender
T5	Guaiacol	1095	0.05	0.03	0.00	0.00	0.06	0.02	0.01	0.01	***	GC-MS GC-FID	3	smoky, clove-like
T6	α-Terpineol	1171	0.14	0.21	0.26	0.23	0.00	0.09	0.19	0.12	***	GC-MS	280–350	pine, terpene, lilac, citrus
T7	Myrcene	1160	0.02	0.19	0.07	0.12	0.05	0.08	0.11	0.13	***	GC-MS GC-FID	13	slightly spicy, peppery, earthy
T8	(-)-ß-citronellol	1229	0.02	0.05	0.04	0.02	0.02	0.06	0.05	0.03	***	GC-MS GC-FID	na	floral, citronella, rose, leafy, oily petal
T9	Citral	1240	0.03	0.07	0.14	0.02	0.02	0.05	0.04	0.04	**	GC-MS GC-FID	28–120	lemon, sweet, fresh citrusy lemon fatty–aldehydic
T10	Geraniol	1258	0.00	0.08	0.11	0.15	0.00	0.12	0.15	0.19	**	GC-MS GC-FID	75	rose, metallic, fresh green, floral
T11	Eugenol	1326	5.88	9.81	8.56	9.05	4.26	9.55	8.89	8.49	***	GC-MS GC-FID	6–30	spicy clove aroma
T12	β-Damascenone	1359	0.93	0.47	0.87	1.24	0.35	0.54	1.81	0.84	***	GC-MS	4–7	persistent fruity, floral; strawberry− and rose-notes, tobacco
T13	Methyleugenol	1408	0.06	0.03	0.03	0.02	0.02	0.05	0.38	0.16	***	GC-MS GC-FID	na	sweet, fresh, warm, spicy, clove, carnation, cinnamon
T14	(*E*)*-*β-Famesene	1458	0.18	0.02	0.01	0.03	0.01	0.04	0.08	0.06	***	GC-MS	0.01	woody
T15	(*Z*,*E*)-α-Farnesene	1480	0.07	0.01	0.01	0.01	0.01	0.02	0.02	0.00	***	GC-MS	-	-
T16	β-ionone	1490	0.09	0.00	0.00	0.00	0.03	0.04	0.00	0.00	***	GC-MS GC_FID	0.007	sweet, floral, violet, raspberry, woody, fruity–floral
T17	α-Farnesene	1494	0.34	0.01	0.01	0.02	0.07	0.08	0.08	0.01	***	GC-MS	na	woody, green, vegetative
T18	2,3-Dihydrofarnesol	1696	3.63	0.12	0.11	0.24	0.01	0.49	1.31	1.37	***	GC-MS	-	-
T19	Farnesol	1702	7.09	0.28	0.17	0.34	0.14	1.11	3.33	0.85	***	GC-MS	1	sweet
T20	Nerolidol	1552	1.62	0.09	0.18	0.27	0.06	0.29	1.04	0.29	***	GC-MS	10	woody, spicy
T21	Isoeugenol	2250	0.68	0.70	0.86	2.47	0.74	0.76	1.98	1.56	***	GC-MS GC-FID	10	sweet, spicy, clove, woody, carnation, floral
	Other compounds													
O1	1,1-diethoxy-ethane	720	55.35	283.4	334.93	232.38	156.96	50.61	53.61	60.45	***	GC-MS	na	sweet, cream
O2	1,1-diethoxy-propane	814	0.00	0.22	0.39	0.19	0.28	0.00	0.00	0.00	***	GC-MS	na	cabbage
O3	1,1-diethoxy-butane	872	0.19	0.75	0.86	0.31	0.95	0.54	0.13	0.22	***	GC-MS	na	fruity, fatty
O4	1,1-diethoxy-pentane	966	0.51	3.23	3.10	1.16	24.58	1.08	0.73	0.68	***	GC-MS	-	-
O5	*n*-Decanoic acid	1360	1.74	0.06	0.08	0.00	0.05	0.00	0.00	0.00	***	GC-MS	0.0071	fatty

n = 3, na—not significant, displaying the significance at 0.001 ‘***’ 0.01 ‘**’ 0.05 ‘*’’; ^1^ LRI—Linear Retention Index; the amount of components was determined semi-quantitatively by measuring the relative peak area of each identified compound, according to the NIST database, in relation to that of the internal standard. The concentrations of labelled volatile compounds were confirmed based on the published studies [5,8,25,29,30]; * Odour description: Burdock [31]. In general, the application of keeving has contributed to a decrease in the concentration of ethyl, methyl, and some other esters (e.g., ethyl hexanoate, diethyl succinate, ethyl octanoate, ethyl propionate, methyl decanoate, methyl laurate, ethyl stearate, isoamyl lactate, isobutyl octanoate, isopentyl octanoate). It has been shown that sugar metabolism is inhibited in stuck fermentations because the proteins involved in this process are produced at lower levels, so it is observed that higher alcohols dominate the volatile profile over esters. Low initial nitrogen levels reduce the growth rate and biomass formation, resulting in a low rate of sugar catabolism. The lack of a nitrogen source causes an interruption in protein synthesis, resulting in the inactivation of sugar transport. Stuck fermentations have been shown to result in an increased production of higher alcohols and a low production of esters and long chain volatile fatty acids [32]. The total sugar concentration, the relative content of different fermentable sugar sources, and their availability affect ester production [27,33]. Considering the number of esters, this conclusion is consistent with our results. Furthermore, Eleutério dos Santos et al. [34] confirmed that ciders produced with a low nitrogen or amino acid content showed slower fermentation and about 50% less volatile compounds, mainly 3-methyl-1-butanol (isoamyl alcohol) and esters.

## Data Availability

Data are contained within this article.
